# The development of synthetic biology: a patent analysis

**DOI:** 10.1007/s11693-013-9121-7

**Published:** 2013-08-22

**Authors:** Davy van Doren, Stefan Koenigstein, Thomas Reiss

**Affiliations:** 1 Fraunhofer Institute for Systems and Innovation Research (ISI), Breslauer Straße 48, 76139 Karlsruhe, Germany; 2 Department of Technology Design and Development, University of Bremen, Badgasteiner Str. 1, 28359 Bremen, Germany

**Keywords:** Synthetic biology, Patent analysis, Emerging technologies, Innovation dynamics

## Abstract

In the past decades, synthetic biology has gained interest regarding research and development efforts within the biotechnology domain. However, it is unclear to what extent synthetic biology has matured already into being commercially exploitable. By means of a patent analysis, this study shows that there is an increasing trend regarding synthetic biology related patent applications. The majority of retrieved patents relates to innovations facilitating the realisation of synthetic biology through improved understanding of biological systems. In addition, there is increased activity concerning the development of synthetic biology based applications. When looking at potential application areas, the majority of synthetic biology patents seems most relevant for the medical, energy and industrial sector. Furthermore, the analysis shows that most activity has been carried out by the USA, with Japan and a number of European countries considerably trailing behind. In addition, both universities and companies are major patent applicant actor types. The results presented here form a starting point for follow-up studies concerning the identification of drivers explaining the observed patent application trends in synthetic biology.

**Electronic supplementary material:**

The online version of this article (doi:10.1007/s11693-013-9121-7) contains supplementary material, which is available to authorized users.

## Introduction

Within life sciences, the concept of synthetic biology has gained interest over the past decade due to its believed potential to improve biotechnology-based translational applications (Burbelo et al. 2010). Its transformation from a philosophical construct towards a real discipline has led to awareness among stakeholders in a position to influence or become affected by synthetic biology’s diffusion into society. However, in spite of its expansion over the past decade, a lack of specific market-based studies limits the potential to properly assess synthetic biology’s progress regarding R&D and market penetration attempts.

A number of science and technology indicators can be applied to asses technological progress, including patent statistics (Hinze and Schmoch 2004). Patent indicators can be used for a number of reasons (ibid.). First of all, patent indicators can be considered early signals for future commercial applications. Due to the general underlying costly and time-consuming nature of patent application, patent statistics indicate a certain amount of optimism or expectation concerning described features within patents showing economic potential (Basberg 1987). Secondly, patent analysis can be valuable in analysing technology development in relation to competition between sectors or countries (Liu and Shyu 1997). Thirdly, based on assessments of current technology trends, patent analyses can be used in planning technology oriented national strategies (Abraham and Moitra 2001). Finally, patent analysis data can be used in modelling practices concerning market development of emerging technologies (Ashton and Sen 1988). 

Innovation research based on patent analyses seems to indicate that emerging technologies generate specific patterns. Traditionally, patent development within specific sectors was believed to follow an S-shaped pattern (Liu and Shyu 1997). A more recent study, based on analyses of over 40 different science-based emerging technologies, expanded this pattern by identifying the following phases (Schmoch 2007):^1^

1. An initial start of mainly experimentation and knowledge gain
2. Increased development of R&D once commercial exploitability has been reached
3. Disillusionment of industry as a result of being non-competitive compared to alternative technologies on industrial scale
4. Regained interest of industry, as a result of being competitive compared to alternative technologies on industrial scale
5. Decreasing patent applications as a result of field saturation of the technology

Regarding this model, analysing patent trends of synthetic biology could provide valuable information in what developmental phase synthetic biology could be placed. Furthermore, a patent analysis provides an opportunity to perform cross-country comparisons regarding patent filing dynamics. In addition, a patent analysis could assist in the identification of actors and organisations active in synthetic biology. Finally, the analysis of synthetic biology patents could provide more detailed knowledge concerning the nature, presence and temporal development of translational synthetic biology patent applications. 

The contribution of this paper is twofold. First, a methodological approach is presented for analysing patenting activities in synthetic biology. One of the difficulties in performing patent analyses for emerging technologies relates to their unspecific classification within the international patent classification (IPC). Due to potential ‘group-overlapping’ nature of emerging technology patent content, such patents are in general classified under multiple existing groups. Therefore, patent content analysis is required to identify emerging technology patents within the IPC. This paper presents a methodology, based on both the IPC and patent content, which can be applied to extract patent applications related to synthetic biology. The second contribution of this paper is the identification of patent application trends within synthetic biology. Based on the applied patent analysis approach, the retrieved patent collection has been used to perform several analyses with respect to cross-country comparisons, actor identification and sector relevant patenting dynamics.

## Methods

### This patent analysis contains the following steps:

- *Step 1: Setting technology independent search criteria* Patents can be filed on both national and international level. Search criteria are set to define in which patent office patents will be searched for. In addition, the time-frame is set to limit the years of patent applications that will be included in the final patent search.
- *Step 2: Develop a technology dependent search strategy* The search strategy is developed based on (a) patent content (keywords, keyword-combinations, keyword-strings), and (b) the IPC.
- *Step 3: Retrieve patents* Based on the developed search strategy, international patent databases are queried; retrieved patents are then examined on relevance for the field of synthetic biology.
- *Step 4:* *Analysing patents* Based on the retrieved synthetic biology patent-pool, a number of analyses are made, including (1) comparative cross-country analysis, (2) actor analysis, (3) sector analysis, and (4) content analysis.

### Step 1: Setting technology independent search criteria

#### Patent office

For the patent search, the World Intellectual Property Organization (WIPO) has been set as inclusive filter criteria. The selection of patents filed directly to this international patent office is due to three reasons. First of all, WIPO patents are regarded as being subjected to high application standards. Secondly, the international nature of WIPO patent applications enhances international comparison due to standardised patent filing conditions and criteria,^2^ thereby avoiding the introduction of statistical bias related to national patent office conditions and characteristics.^3^ Finally, patents filed at international patent offices, in comparison to applications at national offices, indicate a relative high economic and global potential of its described invention. The European Patent Office (EPO), another important international patent office, has been left out in order to avoid a potential bias towards European inventors. Since only WIPO patents were retrieved and analysed, the inclusion of potential multiple patent applications within identical patent families was eliminated.

#### Time-scale

Due to the institutionalised structure of patent processing, patent applications are in general published 18 months after the earliest priority date of patent application. This time-lag, in combination with the dated database of the main patent search engine (PATSTAT, database September 2012), the year 2010 is the most recent year with a complete coverage of filed patents. In order to acquire a historic perspective on patent trend development of the synthetic biology field, patents filed in the period 1990–2010 were selected for final analysis.

### Step 2: Develop a technology dependent search strategy

#### Selection of keywords, keyword-strings and keyword-combinations

For synthetic biology, the identification of suitable keywords is problematic. As it is still not agreed among experts how to exactly define synthetic biology, the identification of proper keywords is challenging.

Keywords have been identified, selected and categorised based on an existing framework developed to position synthetic biology within the field of biotechnology.^4^ This three-dimensional biotechnology framework, based on the identification of drivers relevant for historic biotechnology development, explains the emergence of synthetic biology through the interplay of three distinct drivers, being (1) understanding biological systems, (2) technological resolution, and (3) engineering principles. The framework’s rationale has been used as a starting point for finding suitable keywords. The translation of the framework’s rationale into a patent search strategy was executed into three separate parts:

- *Part 1: Knowledge generation and engineering* Keywords that indicate important facets at different levels of biological systems (Young and Alper 2010); (Rollié et al. 2011) were selected; terms for the objects of biotechnology were combined with terms that indicate the realization of the guiding principles relevant in the synthetic biology research area, including the engineering principles of standardisation, decoupling and abstraction.
- *Part 2: Enabling technologies of synthetic biology* Keywords that indicate enabling technologies, that are believed to be crucial for the maturing of synthetic biology or for the realisation of the guiding principles, were selected.
- *Part 3: Applications of synthetic biology* Keywords that indicate potential applications of synthetic biology were selected.

The keywords have been identified and characterised by means of a qualitative literature analysis, including review articles and other secondary documents.

#### Selection of IPC-(sub) classes and (sub) groups

The IPC consists of five different aggregation levels (http://www.wipo.int/classifications/ipc/en/):

- *Level 1* Section; for example *Section C* chemistry and metallurgy
- *Level 2* Class; for example *Class C12* biochemistry; beer; spirits; wine; vinegar; microbiology; enzymology; mutation or genetic engineering
- *Level 3* Subclass; for example *Subclass C12N* micro-organisms or enzymes; compositions thereof
- *Level 4* Group; for example *Group C12N1* micro-organisms, e.g. protozoa; compositions thereof
- *Level 5* Subgroup; for example *Subgroup C12N1/21* bacteria; culture media therefore modified by introduction of foreign genetic material

Descriptions of all sections, (sub) classes and (sub) groups within the IPC have been reviewed for synthetic biology relevance (http://www.wipo.int/classifications/ipc/en/). IPC sections, (sub) classes and/or (sub) groups were included into the strategy when at least one of the three dimensions of the applied synthetic biology framework was identified within their description.

### Step 3: Retrieve patents

Combinations of *selected IPC*-*(sub) classes/(sub) groups* with *obtained keywords (combinations/strings)* were searched for within three patent databases: (1) DERWENT World Patents Index (http://thomsonreuters.com/ products_services/legal/legal_products/a-z/derwent_world_patents_index/), (2) Espacenet (http://worldwide.espacenet.com/advancedSearch?locale=en_EP), and (3) EPO Worldwide Patent Statistical Database (PATSTAT, Version September 2012; see also http://www.epo.org/searching/subscription/raw/product-14-24.html-PATSTAT queries were conducted with Oracle SQL*Plus).

Combinations were searched for in both patent titles and abstracts. Abstracts of search hits were analyzed regarding their relevance to the field of synthetic biology as described by the applied framework. Ambiguous search terms resulting in false positive hits were identified and adjusted accordingly in order to improve the overall result relevance. The PATSTAT database was used to retrieve the final patent-pool that was subsequently used for analyses.

In Table 1, the final search strategy is listed. The strategy is developed in three parts as discussed in *Step 2* of the methodology. Patents have been retrieved based on both the complete strategy and its individual parts.

**Table 1:** Strategy patent analysis synthetic biology, divided into three parts representing the three dimensions of the field. The asterisk (*) is a wildcard character that matches any occurrence of 0 or more characters

Strategy part	IPC classes	Keywords, string and combinations
Strategy part 1: Knowledge generation and engineering—Understanding and engineering tiers of biological systems	B01; C12N, C12P, C12Q, C12S, C40B	*Keywords*: riboswitch* *Strings*: synthetic biology; synthetic amino acid; synthetic base pair; synthetic genome; synthetic genet*; synthetic nucleic acids; synthetic *nucleotide; synthetic sequence; artificial amino acid; artificial base pair; artificial genome; artificial genet*; artificial nucleic acids; artificial *nucleotide; artificial sequence; genetic circuit; signalling pathway; systems biology; metabolic engineering; synthetic protocell; synthetic cell; artificial cell; minimal cell; cell chassis; vesicul* bioreactor; vesicle bioreactor; minimal genome; synthetic gene cluster; synthetic regulatory network; gene circuit design; biological parts; dna assembly; rational protein design; computational protein design; de novo enzyme design; noncanonical amino acid; unnatural amino acid; rna design; rational design; dna origami; rna nanostructure*; dna nanostructure*; gene* switch; synthetic gene network; artificial gene network; genome engineering; gene oscillator; synthetic shRNA; artificial shRNA; heterologous nucleic acid; biological circuit *Combinations*: molecular machine AND protein; molecular machine AND bio; RNA AND computational design; RNA AND rational design
Strategy part 2: Enabling technologies for synthetic biology	B01; C12N, C12P, C12Q, C12S, C40B	*Keywords*: cad; cam; microfluidics *Strings*: design platform; computer aided design; systems biology model*; metabolomic* model*; transcriptomic* model*; protein folding model*; protein folding prediction; RNA folding model*; RNA folding prediction; multiplex ligation; multiple amplification; dna synthesis; gene synthesis *Combinations*: multiplex AND genome; multiplex AND gene
Strategy part 3: Applications of synthetic biology	C12N; C12P; C12Q; C12S; C40B	*Combinations*: smart material AND bacter*; fuel AND bacter*; energy AND bacter*; medicine AND bacter*; photosynth* AND bacter*; nano* AND bacter*; nano AND bacter*; industr* AND bacter*; remediation AND bacter*; smart material AND microbio*; fuel AND microbio*; energy AND microbio*; medicine AND microbio*; photosynth* AND microbio*; nano* AND microbio*; nano AND microbio*; industr* AND microbio*; remediation AND microbio*; smart material AND microbia*; fuel AND microbia*; energy AND microbia*; medicine AND microbia*; photosynth* AND microbia*; nano* AND microbia*; nano AND microbia*; industr* AND microbia*; remediation AND microbia*
	C12N; C12P; C12Q; C12S	*Combinations*: environment AND degradation

### Step 4: Analysing patents

#### Selection of countries

In this study, the total amount of filed patents, or growth curve, has been used to estimate the development of the field synthetic biology (1) in general, and (2) a selection of countries (Bengisu and Nekhili 2006). All countries were included in the patent search. Since patents can be filed by multiple applicants from different countries, patents were accordingly weighted regarding the involved nationalities of patent applicants.^5^

In addition, two normalisation calculations were performed. First, in order to correct for country population, the amount of patent application per million capita was calculated (Reiss and Lacasa 2008).^6^ And second, in order to assess the relative growth of synthetic biology patent applications within total WIPO patent applications, the proportion of synthetic biology patents in relation to the total amount of WIPO patent applications was determined. These proportions were accordingly normalised with reference year 1990. Total amounts of WIPO patent applications for the period 1990–2010 were identified by means of PATSTAT query.

#### Analysis of both content and application domains of synthetic biology

In order to gain insight regarding the nature of synthetic biology applications, patents retrieved by means of one specific search strategy part (part 3: applications of synthetic biology) were classified according to 8 different categories:

- Industrial biotechnology
- Medicine
- Energy
- Chemical
- Fuel
- Nanotechnology
- Environmental remediation
- Material

Categorisation has been based on both quantitative analyses and qualitative expert-judgement according to a two-step process. First, a pre-categorisation was made based on the applied search terms. In addition, the final categorisation of patents was made based on patent title and patent abstract. After categorisation, keyword searches related to the established categories were made to categorise the individual retrieved patents.

#### Analysing actors in synthetic biology

Based on the retrieved patent-pool, synthetic biology patent applicants have been identified. Weighted patent applications were used to identify the top-20 most active patent filers. These major applicants were categorised to obtain insights concerning their institutional background. The following applicant categorisation has been made:

- Company
- University and college
- Individual
- Research institute

#### Analysis of synthetic biology patents in the IPC

Retrieved patents have also been analysed according to how they have been placed within the IPC. As a result of how the IPC is managed and updated, emerging technologies do often not have a dedicated location within this system. In order to obtain insights regarding patentable domains relevant for synthetic biology, the IPC locations of retrieved patents were analysed.

## Results

### Global synthetic biology activity

**Fig. 1:**
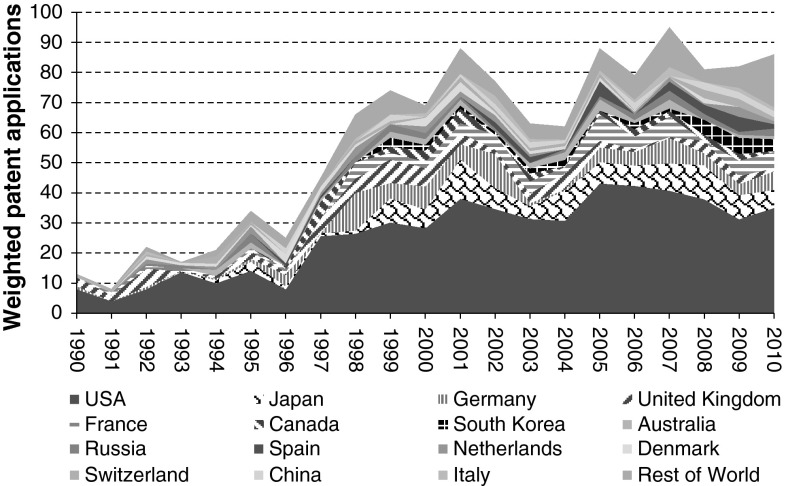
Trend of patent applications in the period 1990-2010: Top-15 countries and rest of world

**Fig. 2:**
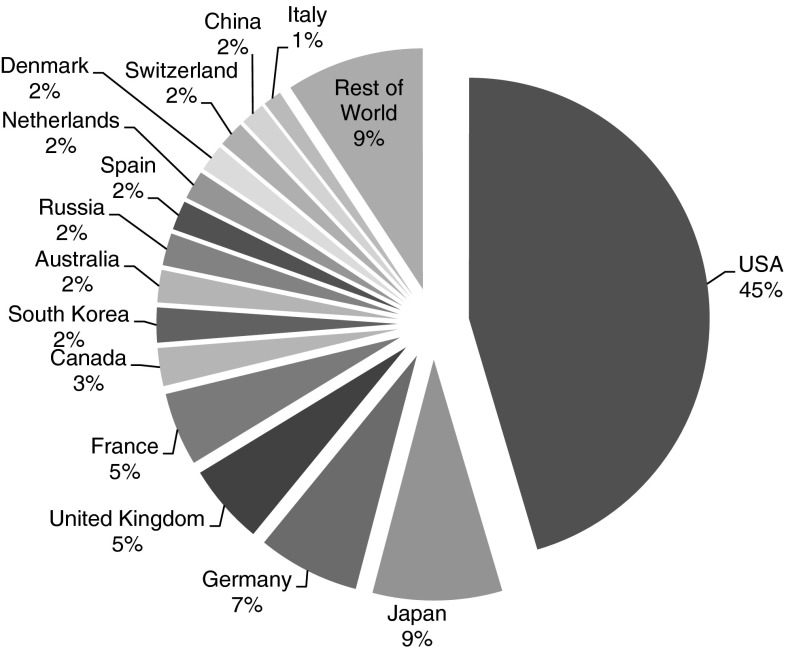
Patent applications for synthetic biology: Share of top-15 countries and rest of world

Based on the applied patent analysis methodology, a total of 1,195 patents was retrieved (Figs. 1, 2). The number of retrieved patents per year is low, not exceeding 95 patents per year. Nevertheless, the analysis shows a clear increase in filed patents over the last 20 years. In 1990, only 13 patents were filed, whereas in 2010 a total of 86 patents were filed. The maximum of patents filed in one year was 95 in 2007, whereas the minimum of patents filed in one year was 8 in 1991.

The USA shows most activity in synthetic biology patent filing, showing an increase from 8.0 (1990) to a total of 35.1 (2008) patents filed by USA applicants. Over the whole analysed period, USA applicants filed 542.6 of the 1,195 retrieved patents. Trailing behind the USA, Japan (103.8 patents) and Germany (81.6 patents) show also significant patent filing activity. Germany (from 1 patent in 1990 to 6.2 patents in 2010) and Japan (from 0 patents in 1990 to 6.0 patents in 2010) also show the largest relative increase in patent application. Other countries showing considerable activity (at least more than 50 patents in 1990–2010) are United Kingdom (64.2 patents) and France (58.8 patents).

**Fig. 3:**
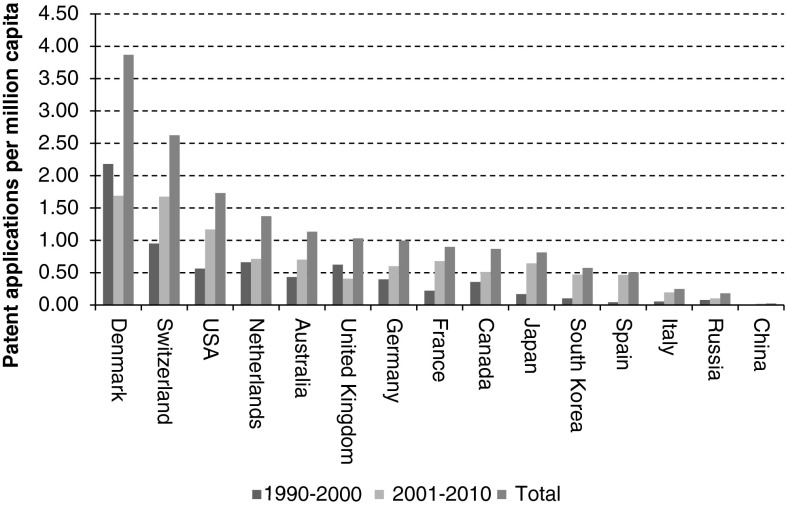
Synthetic biology patent applications of top-15 countries per million capita; both total and time period shares (1990–2000 & 2001–2010) are given

Relative to country population size, minor shifts regarding country ranking with respect to synthetic biology patent applications are observed (Fig. 3). Although the USA still ranks high, it is overtaken by the relative low population European countries Denmark and Switzerland regarding the highest density of synthetic biology patent applicants. Other relative low population countries including Australia and the Netherlands also rank higher, whereas relative high population countries including Japan and Russia rank considerably lower.

**Fig. 4:**
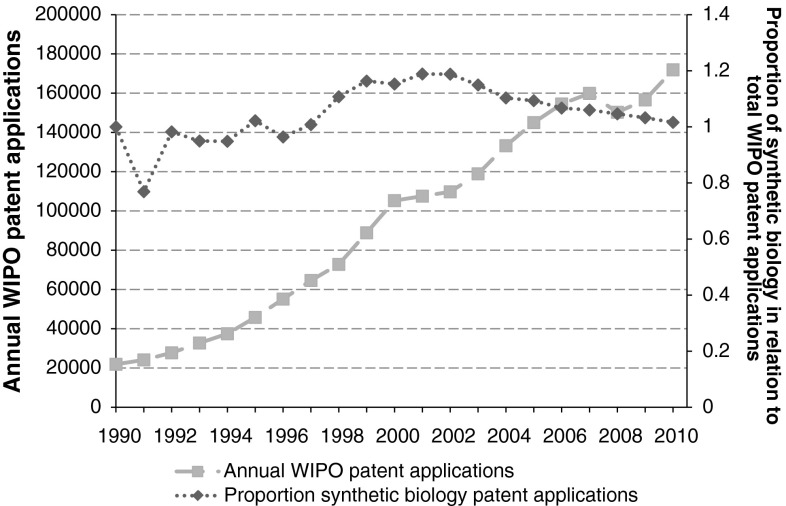
Annual WIPO patent applications and the proportion of synthetic biology patent applications within. Normalisation reference year is 1990

When analysing the proportion of synthetic biology patent applications in relation to total annual WIPO patent applications, we observe an increase of proportion in the period 1991–2001 (Fig. 4). From 2002 onwards, this proportion decreases again towards a comparable proportion of synthetic biology patent applications with respect to the reference year 1990.

### Nature of synthetic biology trends

**Fig. 5:**
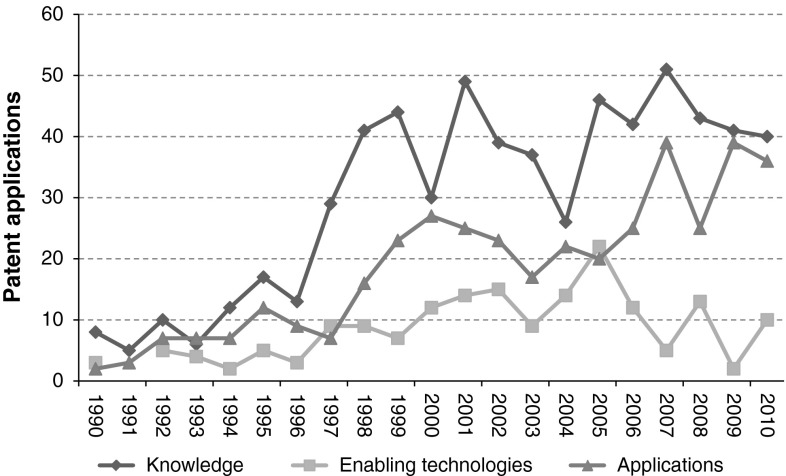
Overview of individual strategy parts: (part 1) knowledge and engineering of synthetic biology, (part 2) enabling technologies of synthetic biology, and (part 3) applications of synthetic biology (part 3)

Besides the complete strategy, parts of the strategy have been used for searches reflecting the development of the different dimensions of synthetic biology (Fig. 5).

The patenting trend related to *knowledge and engineering of synthetic biology (part 1)* shows much similarity in comparison with the overall strategy. The overall trend for this strategy part is increasing from 8 patents filed in 1990 to 40 patents filed in 2010. The maximum amount of patents filed is 51 in 2007; the minimum amount of patents filed is 5 in 1991. It is apparent that research regarding improved understanding of biological systems has not decreased in importance over the last 20 years. This indicates that basic research is still highly important to develop the synthetic biology field.

Patent applications regarding *enabling technologies of synthetic biology* *(part 2)* show a somewhat different pattern. In general, patent filing activity shows an increase from 3 (1990), with an intermediate peak in 2002 (15 patents), to 22 patents being filed in 2005. From 2005 onwards, there is a decline towards 2 patents filed in 2009. However, patent application activity regarding synthetic biology enabling technologies increases again in 2010 (10 patents).

The patenting trend regarding *applications of synthetic biology (part 3)* indicates increasing patent filing activity over the whole analysed period. First signs of considerable activity start to emerge around 1997. From 1997 onwards, patent filing activity increases towards a peak of 39 patents in 2009.

### Synthetic biology actors

**Fig. 6:**
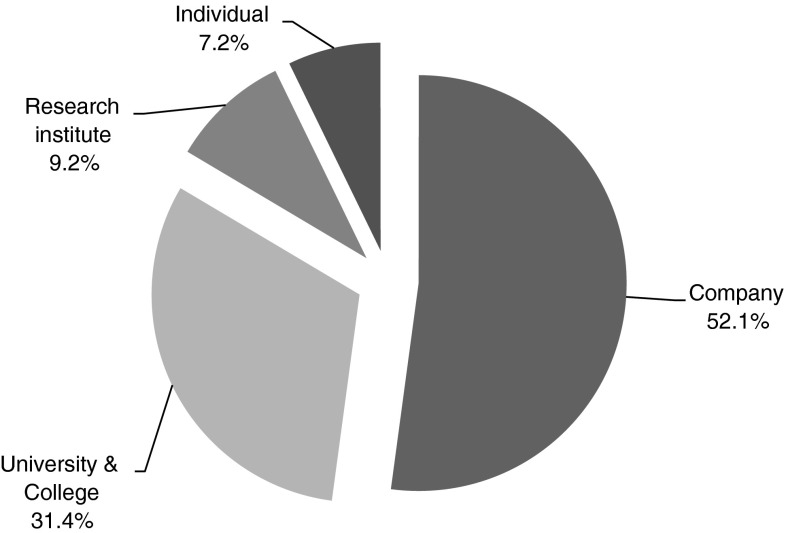
Institutional backgrounds of top-20 synthetic biology patent applicants

A total of 3,988 different applicants were involved in filing the 1,195 retrieved synthetic biology patents. Of these 3,988 applicants, the 20 most active applicants filed more than 2 patents (weighted value with respect to the amount of applicants per patent application) (Table 2; Fig. 6). Concerning the institutional background of patent applicants, the majority originates from companies.

**Table 2:** Overview patent applicants

Applicant	Amount of filed patents (weighted)	Institutional background
The regents of the University of California	9.1	University and college
Hybridon, Inc.	6.6	Company
Yale University	4.8	University and college
Novo Nordisk a/s	4.2	Company
Zeneca Limited	4.0	Company
The Scripps Research Institute	3.9	Research institute
Cellectis	3.9	Company
Agritope, Inc.	3.3	Company
Avigenics, Inc.	3.2	Company
Board of Regents, the University of Texas system	3.1	University and college
President and Fellows of Harvard College	3.0	University and college
Schultz, Peter, G.	2.8	Individual
Cornell Research Foundation, Inc.	2.7	Company
Fred Hutchinson Cancer Research Center	2.6	Research institute
Ronald Breaker R.	2.3	Individual
Dsm ip Assets B.V.	2.3	Company
Genencor International, Inc.	2.2	Company
Degussa AG	2.2	Company
Baylor College of Medicine	2.2	University and college
Novozymes A/S	2.1	Company

The most active synthetic biology applicant is the University of California, holder of the synthetic biology institute in Berkeley. An equally active synthetic biology patent applicant is Hybridon, a biotechnology company based in Cambridge (Massachusetts). Hybridon, who merged with Idera Pharmaceuticals in 2004, concentrates on the development of therapeutics and diagnostics using synthetic DNA. Most active individual patent applicant in the period 1990–2010 was Peter Schultz, who is conducting basic research related to molecular biological building blocks at the Scripps Research Institute in San Diego, California. Ronald Breaker, another active individual patent applicant, performs fundamental research at Yale University in New Haven concerning structures and functions of RNA and DNA molecules.

### Synthetic biology application areas

Many of the retrieved patents have a strong focus towards both engineered biological parts and the methodology to analyse and produce these. Topics of such focus include DNA synthesis and sequencing methods, evolutionary approaches to construct artificial nucleotide sequences, the production of selective growth cultures, understanding of processes related to cell metabolism, the development of test-systems, and methods related to multiplication, expression and nucleic acid modelling.

**Fig. 7:**
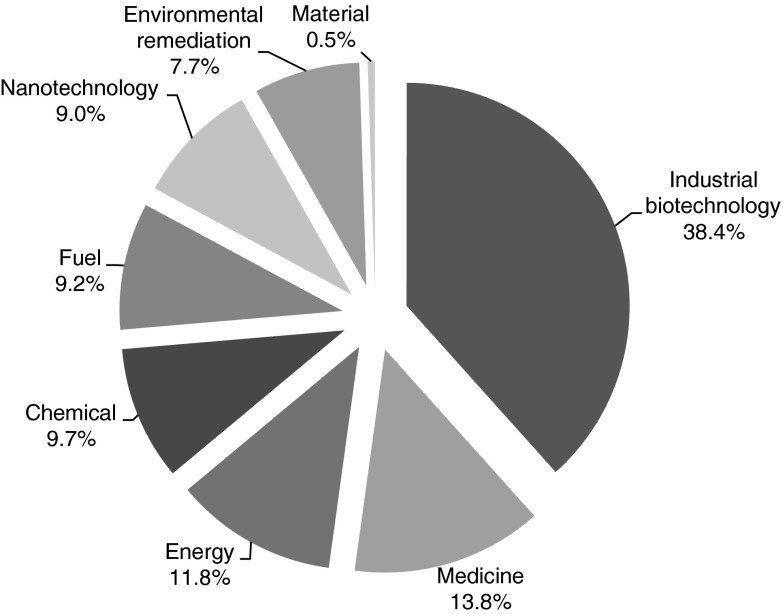
Division of synthetic biology patent applications, in the period 1990–2010, according to their application area. Based on *part 3 applications of synthetic biology* strategy

When looking at relevant application areas regarding synthetic biology patents (strategy *part 3 applications of synthetic biology*), the large majority seems most relevant for industrial biotechnology (38.4 % of in total 391 patents) (Fig. 7). A patent share of 13.8 % seems most relevant for the medical domain. One focus of patents related to medical application includes neural DNA-injection and the production of potentially active pharmaceutical components for a wide variety of medical indications, including nucleic acids, peptides and proteins. Another focus of medical patent applications concerns the unravelling of disease mechanisms and the role of signal transduction therein. Patents relevant for energy production (11.8 %) seem to focus on dominant themes including hydrogen production, photosynthesis process enhancements and feedstock production improvements.

**Fig. 8:**
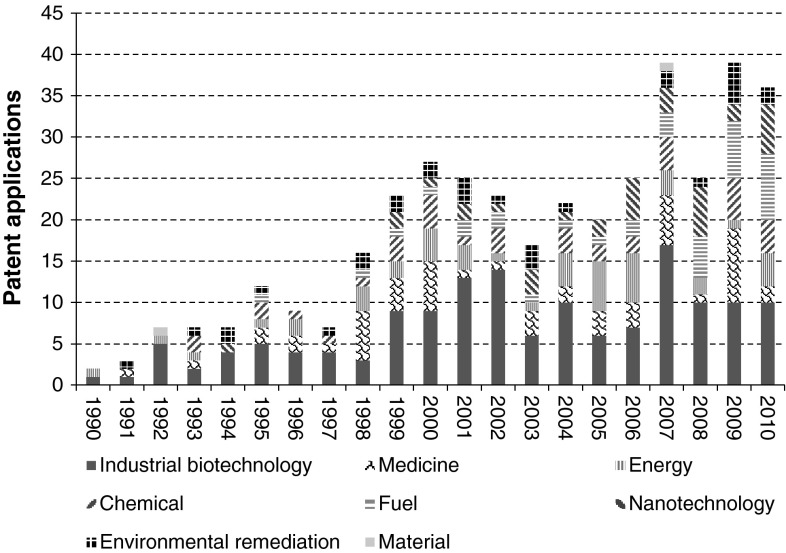
Chronological overview of the absolute amount of patent applications over different application domains. Based on strategy *part 3 applications of synthetic biology*

Concerning the chronological development of synthetic biology patents, it seems that especially application domains related to fuels, nanotechnology and environmental remediation have received increased attention over the past decades (Fig. 8).

### IPC classification of synthetic biology patents

**Fig. 9:**
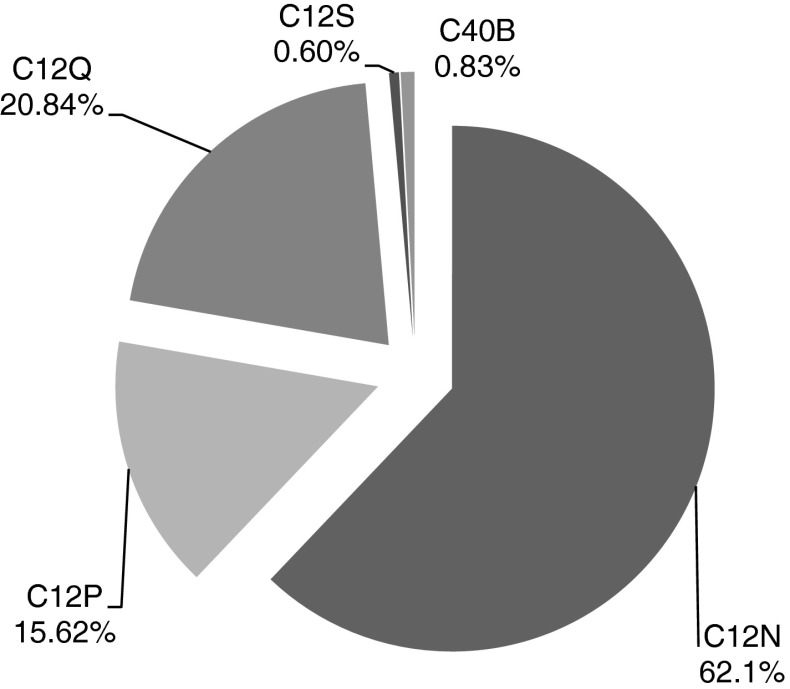
Relative share of synthetic biology patents regarding the international patent classification (IPC)

**Fig. 10:**
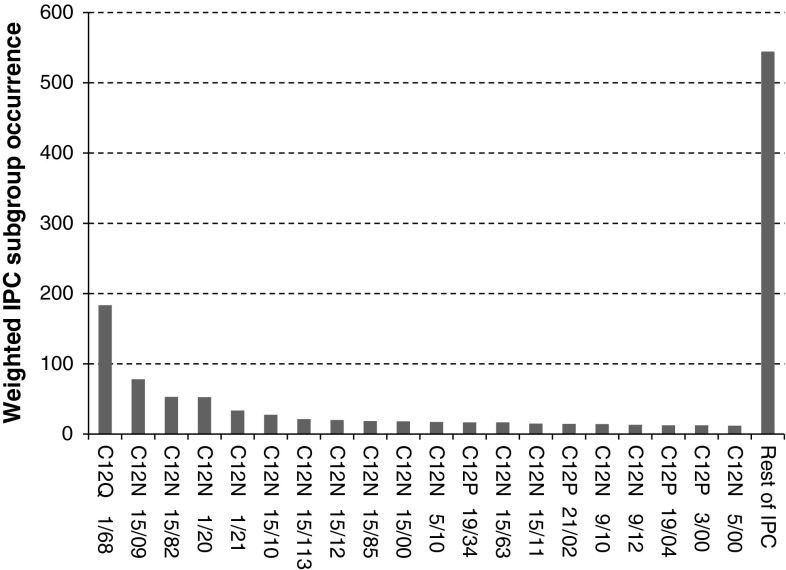
Amount of weighted entries of the top-20 IPC-classes

Class C12 (*Biochemistry*) is by far the most prominent class in which synthetic biology patents are being categorised (Fig. 9; Table 3). Concerning this categorisation, C12Q 1/68 (measuring or testing processes involving nucleic acids) is the most dominant IPC subgroup within the C12 class (Fig. 10).

**Table 3:** Overview of IPC subclasses and top-20 IPC subgroups regarding retrieved synthetic biology patent applications

IPC	Title
*IPC subclass*	
C12N	Micro-organisms or enzymes
C12P	Fermentation or enzyme-using processes to synthesise a desired chemical compound or composition or to separate optical isomers from a racemic mixture
C12Q	Measuring or testing processes involving enzymes or micro-organisms
C12S	Processes using enzymes or micro-organisms to liberate, separate or purify a pre-existing compound or composition
C40B	Combinatorial chemistry
*IPC subgroup*	
C12N 1/20	Micro-organisms—bacteria
C12N 1/21	Micro-organisms—bacteria modified by introduction of foreign genetic material
C12N 5/00	Undifferentiated human, animal or plant cells
C12N 5/10	Undifferentiated human, animal or plant cells—cells modified by introduction of foreign genetic material
C12N 9/10	Enzymes
C12N 9/12	Enzymes—transferases
C12N 15/00	Mutation or genetic engineering
C12N 15/09	Mutation or genetic engineering—recombinant DNA-technology
C12N 15/10	Mutation or genetic engineering—recombinant DNA-technology; processes for the isolation, preparation or purification of DNA or RNA
C12N 15/11	Mutation or genetic engineering—recombinant DNA-technology; DNA or RNA fragments
C12N 15/12	Mutation or genetic engineering—recombinant DNA-technology; DNA or RNA fragments; Genes encoding animal proteins
C12N 15/63	Mutation or genetic engineering—recombinant DNA-technology; introduction of foreign genetic material using vectors
C12 N 15/82	Mutation or genetic engineering—recombinant DNA-technology; introduction of foreign genetic material using vectors; Vectors or expression systems specially adapted for eukaryotic hosts; for plant cells
C12N 15/85	Mutation or genetic engineering—recombinant DNA-technology; Introduction of foreign genetic material using vectors; vectors or expression systems specially adapted for eukaryotic hosts; for animal cells
C12N 15/113	Mutation or genetic engineering—recombinant DNA-technology; DNA or RNA fragments; non-coding nucleic acids modulating the expression of genes
C12P 3/00	Preparation of elements or inorganic compounds except carbon dioxide
C12P 19/04	Preparation of compounds containing saccharide radicals—polysaccharides
C12P 19/34	Preparation of compounds containing saccharide radicals—preparation of nitrogen-containing carbohydrates; N-glycosides; nucleotides; polynucleotides
C12P 21/02	Preparation of peptides or proteins—having a known sequence of two or more amino acids
C12Q 1/68	Measuring or testing processes involving enzymes or micro-organisms—involving nucleic acids

Both classifications and titles of subclasses/subgroups are given

## Discussion

### Main findings

This analysis does not intend to provide full explanations of the observed trends in patent development within the field of synthetic biology. In order to compare the activities of different countries in more detail, a deeper analysis into drivers of national innovation performance is required. Furthermore, it is not clear to what extent a patent analysis suffices in giving a comprehensive representation of synthetic biology’s development. Patent statistics is only one of many available science and technology indicators that can be applied to assess development of the synthetic biology sector. Accordingly, analyses of the retrieved patent data, including derived conclusions regarding the patent analyses, should be interpreted with care.

Taking these limitations into account, the presented analysis provides some hints towards potential explanations related to the development of the synthetic biology field. One of the main outcomes is the confirmation of the prematurity of synthetic biology in terms of patenting activity. This might indicate that synthetic biology activities are still in an early developmental phase, with still a large focus on exploration through basic research.

Considering the observed patenting trends of the different strategy parts, some possible hypotheses regarding their underlying drivers could be posed. Initial activity in basic research might have fuelled patent development of enabling technologies. Increased focus on the development of these enabling technologies might have been stimulated through a number of large scale research projects. The combined effect of knowledge generation and enabling technologies might have led to a significant increase in developing application oriented patents around 2005 onwards, by both existing organisations and new synthetic biology start-ups.

In general, also in reflection of the different developmental phases of emerging technologies (Schmoch 2007), the field of synthetic biology seems to be in the first phase of mainly experimentation and knowledge gain. Nevertheless, the increasing trend of application oriented patents might be an indication of a gradual transition into the next phase of commercial exploitation.

### Comparing countries

The large contribution of the USA in synthetic biology patent development might be a consequence of USA’s history in biotechnology development in general. USA is still by far the most active country concerning the amount of biotechnology firms (Van Beuzekom and Arundel 2009), the development of industrial biotechnology,^7^ and investments in biotechnology-based R&D.^8^ The dominance of the USA is also reflected in patent applications of biotechnology in its totality (Reiss and Lacasa 2008).

Outside the USA, there is a large impact made by Japan. Other Asian countries showing considerable patent filing activity include South Korea and China. China has already shown signs of large interest in developing this field (Pei et al. 2011); (Zhang et al. 2011). India, although not very active yet with respect to synthetic biology patent filings, has shown the largest relative increase in biotechnology patents granted in the period 2000–2009 (OECD 2010).

It could be argued that the general trailing of European countries behind USA regarding synthetic biology patent applications may be caused by Europe’s strong historic position within the chemical sector. However, such orientation does not need to obstruct synthetic biology developments. This patent analysis has shown that Germany and the United Kingdom, both holding the largest chemical companies in Europe,^9^ are also the most active European countries with regard to synthetic biology patent applications. The potential of biotechnology to produce chemicals, thereby decreasing the dependence on fossil sources for their production, might have been an incentive for these countries to invest in synthetic biology related activities. Nevertheless, follow-up research is required to validate the correctness of such speculation.

### Applications of synthetic biology

An increasing trend regarding application related synthetic biology patents is observed. A large share of these patents seems to relate to industrial biotechnology. Besides this focus on industrial biotechnology, the interest of synthetic biology in the medicine area is not surprising. The focus on developing new pharmaceutics reflects present expectations concerning the potential of biotechnology in general. This might explain the contribution of the UK in synthetic biology patents, having shown a historic trend of applying biotechnology in developing the pharmaceutical sector (Boyle 2011).

There is also considerable interest in patents related to fuel and energy production. This trend might have been driven by renewed interest in fossil source independency, stimulating the use biotechnology for biofuel production. In conjunction with external data about the current production of biofuels within Europe, this application area of synthetic biology might become commercially viable on short term. Large interest from leading countries like Germany, France, Italy and Spain (EUROSTAT 2011) might indicate a certain orientation of these countries towards the supply, transformation and consumption of biofuels.

### Institutional context

When looking at the actors involved in synthetic biology patenting, it can be observed that mainly leading universities, relatively new synthetic biology specific companies and a number of multinational companies are active. The involvement of novel innovative synthetic biology companies might have been driving forces for the development of synthetic biology. The involvement of longer existing large multinational companies could be explained with the current focus of synthetic biology applications in the areas of medicine and chemistry, sectors which are dominated by traditional large companies. The patenting activity of these large companies could also indicate their high expectations concerning the future potential of synthetic biology.

### Limitations of the study

Within this study, there are a number of limitations that need to be mentioned.

One limitation lies within the relative low amount of retrieved patents. Although this amount could reflect the developmental state of the field, it introduces a large influence of noise, hampering interpretation of the strategy’s output. It is therefore difficult to judge to what extent fluctuations in the observed trends are real changes of activity related to synthetic biology, or merely artefacts of present noise.

A second limitation of this analysis is the lack of qualitative indices for patent strength, including citation-indices or regression models. Citation-indices can be used to (1) indicate the importance of a patent for other patents, or (2) to describe the relationships between patents or their developers through the visualisation of citation networks (Brinn et al. 2003). Regression models can be used to analyze the relationship between (1) patent development, and (2) R&D spending at the firm level (Wang et al. 1998). However, the relative low amount of patent application of synthetic biology limits the extent statistical tools can be used to create a more robust picture.

A third limitation concerns the development of the overall strategy. Although the applied search criteria have arguably facilitated the retrieval of synthetic biology relevant patents, they also limit the absolute scope of a patent search. As a result, synthetic biology relevant patents that are hard to identify by means of specific search criteria might have been unintentionally excluded from the analysis. Although this is a general limitation within the applied methodological approach, it limits the abstraction of conclusions regarding the absolute developmental state of the synthetic biology field. In addition, due to the heterogeneous collection of present synthetic biology perceptions, defining a strategy that could be agreed upon unanimously is challenging. This issue was addressed in the strategy by integrating the suggested three fundamental drivers for the development of synthetic biology. Although the applied framework seems to be a valid representation of current discussions concerning delineation of the synthetic biology, this analysis should be regarded as a particularly and temporary interpretation of this emerging field.

A final limitation regards to what extent the observed patent dynamics represent comprehensively and completely research, development and market activities for emerging technologies. Especially in the synthetic biology domain, there is considerable discussion regarding the appropriateness of alternative strategies with regard to intellectual property management (e.g., see Henkel and Maurer 2009). Standardisation, open source and collaboration oriented business model innovation might already influence the role and value of synthetic biology patent applications. Therefore, the results obtained through this study should be interpreted as a partial representation regarding synthetic biology developments.

### Further research

The main aim of this research was to provide a methodological approach to identify synthetic biology patents. Based on the developed methodological approach, identified patent applications were analysed to enable insights regarding the dynamic, nature and actor specificity within the field of synthetic biology. These dynamics could serve as a foundation for future research in explaining and identifying both technological and non-technological factors driving the observed synthetic biology patent activity.

The provided analysis provides some initial indications and nation-based trends concerning the development of the emerging field synthetic biology. However, this paper does not provide answers for all observed phenomena within the analysis. Further research is required to provide explanations concerning the nature of underlying drivers for these phenomena and the validity of the proposed potential explanations.

And finally, the analysis of technology trends alone cannot incorporate the organizational and political scenarios that will influence the development and direction of future technologies (Daim et al. 2006). A deeper investigation into potential drivers of innovation, including processes underlying market regulation and social acceptance, could facilitate deeper insight of key actor behaviour. Such analyses could provide improved understanding with respect to the observed patent trends in synthetic biology.

## Electronic supplementary material

Below is the link to the electronic supplementary material.

Supplementary material (XLSX 673 kb)

## Footnotes

1 The resulting pattern was labelled as representing "double-boom cycles".

2 Patent applications at WIPO fall under the PCT (Patent Classification Treaty). The PCT is an international treaty, administered by the World Intellectual Property Organization (WIPO), between more than 140 Paris Convention countries (http://www.wipo.int/pct/en/faqs/faqs.html), including the most active European, Asian and North- and South-American countries.

3 One example of such bias is the exclusion of patents filed in distant languages using different symbols (Asian, Russian, Arabic).

4 Forthcoming book chapter for the Edward Elgar Series on Science, Technology, Innovation and Entrepreneurship, in progress. See also van Doren and Reiss 2012.

5 For example, when patent x was filed by applicant A from USA and applicant B from Germany, both applicants receive 1/2 of the co-applied patent. As a result, final analyses regarding patent amounts can contain fractions.

6 Source country population data: http://www.nationsonline.org/oneworld/population-by-country.htm.

7 Published by FAZ (2008). Used sources include Coston Consulting Group (BCG), Bio Deutschland, EMEA, Ernst & Young, European Commission, FAZ-Archive, Companies and VFA.

8 Published by Ernst and Young (2010). The pharmaceutical industry in figures.

9 Based on data derived from Amadeus (2011): see https://amadeus.bvdinfo.com/version-2012104/home.serv?product=amadeusneo.
